# Loss of O-GlcNAcase catalytic activity leads to defects in mouse embryogenesis

**DOI:** 10.1016/j.jbc.2021.100439

**Published:** 2021-02-19

**Authors:** Villő Muha, Florence Authier, Zsombor Szoke-Kovacs, Sara Johnson, Jennifer Gallagher, Alison McNeilly, Rory J. McCrimmon, Lydia Teboul, Daan M.F. van Aalten

**Affiliations:** 1 Division of Gene Regulation and Expression, School of Life Sciences, University of Dundee, Dundee, UK; 2 The Mary Lyon Centre, MRC Harwell Institute, Oxfordshire, UK; 3 Division of Molecular & Clinical Medicine, University of Dundee, Dundee, UK; 4 System Medicine, School of Medicine, University of Dundee, Dundee, UK

**Keywords:** *O*-GlcNAcylation, *O*-GlcNAcase, perinatal lethality, mouse genetics, *in vivo* imaging, microcomputed tomography, glycobiology, development, embryo, AD, Alzheimer's disease, CDG, congenital disorder of glycosylation, *Cp*OGA, clostridium perfringens O-GlcNAcase, HAT, histone acetyltransferase, ID, intellectual disability, mESC, mouse embryonic stem cell, microCT, microcomputed tomography, OGA, O-GlcNAcase, OGT, O-GlcNAc transferase

## Abstract

*O*-GlcNAcylation is an essential post-translational modification that has been implicated in neurodevelopmental and neurodegenerative disorders. *O*-GlcNAcase (OGA), the sole enzyme catalyzing the removal of *O*-GlcNAc from proteins, has emerged as a potential drug target. OGA consists of an N-terminal OGA catalytic domain and a C-terminal pseudo histone acetyltransferase (HAT) domain with unknown function. To investigate phenotypes specific to loss of OGA catalytic activity and dissect the role of the HAT domain, we generated a constitutive knock-in mouse line, carrying a mutation of a catalytic aspartic acid to alanine. These mice showed perinatal lethality and abnormal embryonic growth with skewed Mendelian ratios after day E18.5. We observed tissue-specific changes in *O*-GlcNAc homeostasis regulation to compensate for loss of OGA activity. Using X-ray microcomputed tomography on late gestation embryos, we identified defects in the kidney, brain, liver, and stomach. Taken together, our data suggest that developmental defects during gestation may arise upon prolonged OGA inhibition specifically because of loss of OGA catalytic activity and independent of the function of the HAT domain.

*O*-GlcNAcylation is a dynamic co-/post-translational modification of serine/threonine residues with *N*-acetyl-d-glucosamine, responsible for modulating cellular functions, such as translation (1), protein stability (2, 3), and subcellular localization of proteins (4, 5). *O*-GlcNAcylation is dependent on the nutrient flux through the hexosamine biosynthetic pathway and coordinates transcription (6, 7, 8, 9) and differentiation (10, 11, 12) according to the metabolic status of the cell and organism. The entire *O*-GlcNAcome, over 4000 proteins, is established by *O*-GlcNAc transferase (OGT) (13) that catalyzes the addition of *N*-acetylglucosamine and *O*-GlcNAcase (OGA) that mediates the removal of the modification (14).

*O*-GlcNAcylation and the *O*-GlcNAc cycling enzymes are critical for normal development in several organisms. In *Drosophila*, *ogt*, also known as *super sex combs* (*sxc*), is essential for viability and correct segment identity specification (15, 16). *Ogt* KO leads to impaired embryonic growth and cell viability in zebrafish (17). Deletion of either *Ogt* or *Oga* is lethal in mice (18, 19, 20). In mammals, OGT and OGA are ubiquitously expressed, and they regulate the development of several tissues (21, 22, 23). *O*-GlcNAcylation modification is particularly abundant in the mammalian brain (24, 25, 26), and both *O*-GlcNAc cycling enzymes are important for normal development and function of the central nervous system (27, 28, 29). Very recently, missense *OGT* mutations have been identified and characterized in patients affected with intellectual disability (ID) in association with developmental delay and brain anomalies (30). ID is a neurodevelopmental disorder that affects 1 to 2% of the population and is characterized by impaired cognitive function and adaptive behavior (31). Phenotypic characterization of several *OGT* variants gave rise to a new syndrome of congenital disorder of glycosylation named OGT-CDG; however, the mechanisms underlying the patient brain phenotypes remain unknown (30). Interestingly, a reduction of OGA expression was observed in several mouse embryonic stem cell (mESC) lines carrying OGT-CDG mutations (32, 33, 34, 35), suggesting that altered OGA expression may be associated with reduced OGT activity in some mutations and might contribute in part to the neuronal phenotype in these patients. OGA has been shown to contribute to proper brain function using several animal models. In flies, loss of OGA activity affects cognition and synaptic morphology (36). Viable heterozygous *Oga*^*+/−*^ mice exhibit learning and memory impairment (29). In addition, a genome-wide association study using 14 independent epidemiological human cohorts has associated SNPs in *OGA* with intelligence and cognitive function (37), suggesting a role of OGA in human cognitive function.

Deregulation of *O*-GlcNAcylation has also been linked to neurodegenerative disease. However, it appears difficult to define a general neuroprotective or neurodegenerative role for *O*-GlcNAcylation. *O*-GlcNAc protein levels are found reduced in Alzheimer's disease (AD) while being increased in Parkinson's disease postmortem human brain tissues (38, 39). Many proteins associated with these diseases are *O*-GlcNAcylated, including tau, α-synuclein, and β-amyloid precursor proteins (40). Several studies have shown that reduction of *O*-GlcNAc levels leads to neurodegeneration. For example, the reduction of *O*-GlcNAcylation in forebrain excitatory neurons leads to progressive neurodegeneration associated with neuronal cell death, neuroinflammation, increased levels of hyperphosphorylated tau, and β-amyloid peptides in mice (41). Moreover, it has been shown that *O*-GlcNAc at specific sites reduced α-synuclein aggregation and cell toxicity using synthetic protein methodology (42). However, other studies have also suggested a role of increased *O*-GlcNAcylation in neurodegeneration. Increase of *O*-GlcNAc modification in the mouse hippocampus and neuronal precursor cells is associated with neuronal apoptosis (24, 43). In addition, elevation of *O*-GlcNAc levels through pharmacological OGA inhibition causes an increase of α-synuclein accumulation and a decrease of autophagic flux prior to neuronal cell death in rat primary neurons (38). Considering the number of *O*-GlcNAc-modified proteins in the brain, it is likely that the role of this protein modification depends on each specific substrate and the cellular, tissue, and disease contexts.

Nevertheless, emerging evidence suggests that elevation of *O*-GlcNAc levels through pharmacological inhibition of OGA may be a relevant therapeutic strategy for the treatment of AD. OGA inhibition through chronic treatment with OGA inhibitors reduces tauopathy phenotypes in several mouse AD models (44, 45, 46, 47). It has been hypothesized that *O*-GlcNAcylation of tau could prevent its phosphorylation and aggregation (48). Although a reduction in tau hyperphosphorylation and aggregation have been observed after OGA inhibition, it remains uncertain whether it is mediated directly through *O*-GlcNAcylation as increase in tau *O*-GlcNAcylation *in vivo* has only been observed in one study (44). It may be possible that the observed benefits are mediated through other *O*-GlcNAcylated substrates as global elevation of *O*-GlcNAcylation is achieved upon OGA inhibition. Considering that *O*-GlcNAcylation is important in regulating several biological functions, it is imperative to understand the molecular mechanisms and physiological consequences of prolonged OGA inhibition.

Despite the critical role of OGA, our understanding about how OGA activity participates in development and pathological process remains limited. OGA is a 103 kDa multidomain protein (Fig. 1*A*); it consists of an N-terminal *O*-GlcNAc hydrolase catalytic domain with sequence homology to glycoside hydrolase family 84, a middle, mostly disordered, “stalk” domain and a C-terminal domain showing sequence homology to GCN5-related histone acetyltransferases (HATs) (49). Initially, OGA was reported to possess HAT activity (50), but other studies failed to reproduce this observation (51, 52, 53). OGA most likely contains a pseudo-HAT domain because it lacks key amino acids required for acetyl coenzyme A binding (52) required for histone acetylation. Thus, it has been established that OGA only possesses OGA catalytic activity (52). Phenotypic comparison of *Oga*^*KO*^ and catalytically dead *Oga*^*D133N*^ *Drosophila* alleles, however, revealed differences in behavior and neuronal phenotypes that suggest nonenzymatic functions of the OGA protein backbone (36). These additional functions could be potentially dependent on the HAT domain whose function still remains unknown.

**Figure 1:**
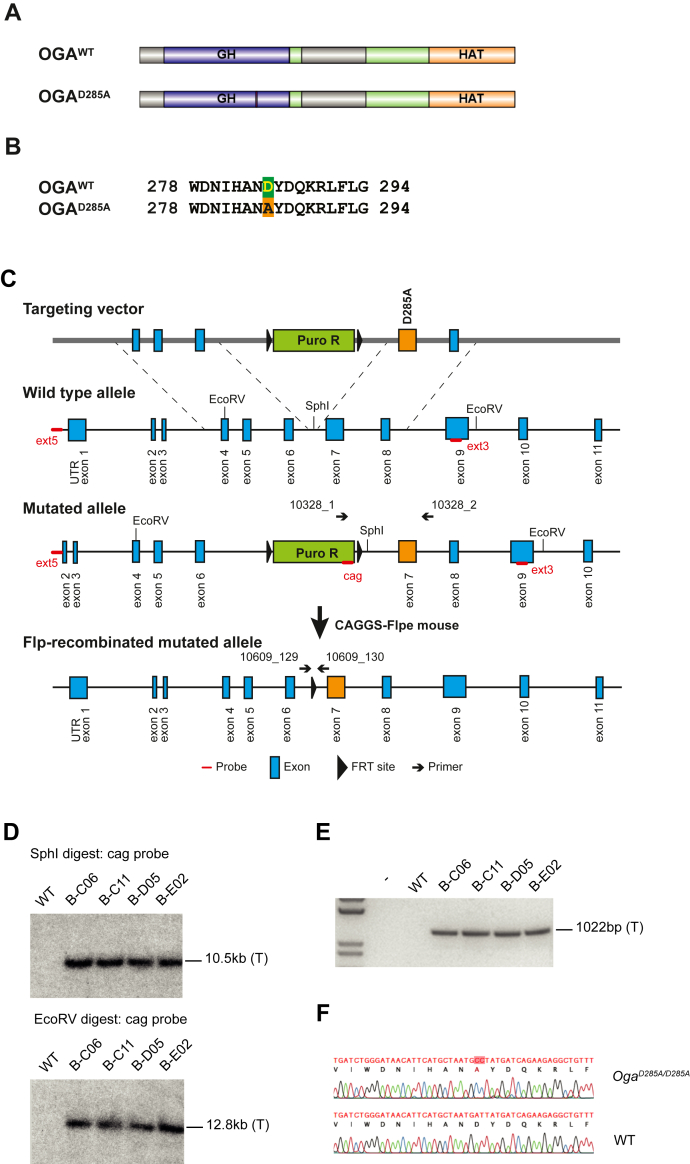
**Generation of the *O*-GlcNAcase catalytically dead *Oga***^***D285A***^**knock-in mouse line.***A*, schematic representation of OGA protein; *purple* glycosyl hydrolase (GH) domain, *green* stalk domain, and *orange* pseudo-histone acetyltransferase domain. Unstructured regions are colored *gray*. The *Oga*^*D285A*^ allele expresses full-length OGA with a single D285A missense mutation in the GH domain (the mutation site is in *orange*). *B*, sequences of OGA^WT^ and OGA^D285A^ 278 to 294 residues highlighting the D285A site are shown in *green*/*orange*. *C*, schematic representation of the ∼10 kb *Oga*^*D285A*^ transgene targeting exon 4 to 8 in the *Oga* gene used to generate *Oga*^*D285A*^ mouse embryonic stem cells (mESCs). Recombinant cell colonies, encoding FRT-flanked puromycin resistance gene, were isolated and injected into blastocysts. Highly chimeric progenies were crossed with Flp deleter (C57BL/6-Tg(CAG-Flpe)2Arte) mice to eliminate puromycin resistance. The locations of the enzymatic restriction sites, primers, and probes are also shown. *D*, southern blot of WT and four targeted mESC clones (named B-C06, B-C11, B-D05, and B-E02) with the cag probe shows correct homologous recombination at the 5’ side (*upper panel*) and 3’ side (*lower panel*) and single integration in all clones. The expected molecular weight bands for the targeted (T) allele are shown. *E*, PCR of WT and four targeted mESC clones shows insertion of the point mutation in all targeted clones. The expected molecular weight band for the targeted (T) allele is indicated. *F*, sequencing of genomic DNA from knock in *Oga*^*D285A/D285A*^ and WT confirms the presence of single D285A point mutation (in *red*) in knock in animal. FRT, flippase recognition target; OGA, *O*-GlcNAcase.

OGA is indispensable for late embryonic development in mammals (19, 54). In two *Oga* KO (*Oga*^*KO*^) mouse models, embryos exhibited a reduction in size, and neonates died within 48 h after birth (19, 54). Although the precise causes of death remain unknown, the lethal phenotype was associated with abnormal lung histology marked with a reduction in prealveolar space in 18.5 dpc embryos (19), hypoglycemia, and impaired glycogen deposition in the liver of neonates (54). Mouse *Oga*^*KO*^ studies using mouse embryonic fibroblasts or liver tissues from homozygous animals assigned these phenotypes to different cellular defects, specifically to mitotic abnormalities, cytokinesis failure (19), and altered metabolic signaling (54, 55). However, it remains unknown if these phenotypes are specifically because of the loss of OGA enzymatic activity or loss of the OGA protein.

To investigate the role of OGA catalytic activity in development and disease, we generated a knock-in *Oga* mouse model (*Oga*^*D285A*^), where a key residue for catalytic activity, D285, responsible for *O*-GlcNAc moiety binding and hydrolysis, is mutated to alanine (56). Therefore, the *Oga*^*D285A*^ animals lack OGA activity while still expressing full-length OGA protein. This genetic strategy can model chronic inhibition of OGA activity in mouse, allowing the evaluation of the long-term effects of OGA inhibition and the investigation of the potential role of the HAT domain of OGA *in vivo*. We show that loss of OGA activity leads to perinatal lethality, organ defects, and tissue-specific disruption in *O*-GlcNAcylation homeostasis in embryos. These results highlight the essential role of the OGA domain during development independently of the HAT domain. Although the use of this model in adult animals is limited because of the lethality observed at the homozygous level, a similar strategy could be used to generate inducible mouse model carrying the *Oga*^*D285A*^ mutation that will allow the future investigation of long-term adverse effects of prolonged OGA inhibition during adulthood *in vivo*.

## Results

### Loss of OGA catalytic activity leads to perinatal lethality

To dissect the role of OGA catalytic activity in mammalian development, we designed an *Oga* constitutive knock-in mouse model where OGA enzymatic activity is abolished (Fig. 1, *A* and *B*). Previous studies have shown that OGA Asp285 (D285) is essential for both *O*-GlcNAc hydrolysis and *O*-GlcNAc binding (56, 57, 58), whereas mutation to alanine causes nearly 100% loss of catalytic efficiency *in vitro* (59). We introduced the OGA D285A mutation into mESCs by homologous recombination (Fig. 1*C*). Correct single integration of the point mutation into mESCs was determined by Southern blotting (Fig. 1*D*) and PCR genotyping (Fig. 1*E*). Clones positive for the mutation were used for blastocyst injections to generate an *Oga*^*D285A*^ mouse line lacking OGA enzymatic activity. DNA from positive offspring was sequenced to confirm the presence of the D285A mutation (Fig. 1*F*).

**Figure 2:**
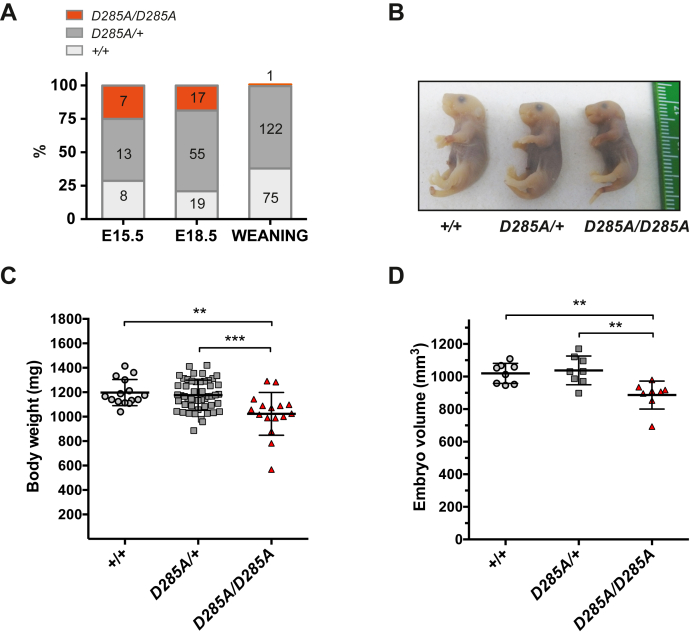
***Oga***^***D285A/D285A***^**mice die perinatally.***A*, survival of *Oga*^*D285A/D285A*^ animals compared with heterozygous and WT littermates at indicated time points. Numbers of each genotypes are shown on the graph. *B*, representative images of WT, heterozygous *Oga*^*D285A/+*^, and homozygous *Oga*^*D285A/D285A*^ 18.5 dpc embryos. *C*, homozygous *Oga*^*D285A/D285A*^ animals were markedly lighter (1023 ± 175 mg, n = 16) than their heterozygous *Oga*^*D285A/+*^ (1176 ± 124 mg, n = 50) and WT (1197 ± 107 mg, n = 15) littermates. Graph shows body weight of 18.5 dpc embryos of each genotype. Mean ± SD, ∗∗*p* = 0.001, ∗∗∗*p* < 0.001, one-way ANOVA with post hoc Tukey's test. *D*, body volume of 18.5 dpc embryos of each genotype. Volumes of 18.5 dpc embryos were determined after microCT scan (n = 8). The *Oga*^*D285A/D285A*^ animals were smaller (887 ± 86 mm^3^) than their heterozygous (1038 ± 88 mm^3^) and WT (1020 ± 62 mm^3^) littermates. (*Oga*^+/+^*versus Oga*^*D285A/D285A*^, ∗∗*p* = 0.008), (*Oga*^*D285A/+*^*versus Oga*^*D285A/D285A*^, ∗∗*p* = 0.003), one-way ANOVA with post hoc Tukey's test.

Previous studies have revealed that mice deficient in OGA protein (*Oga*^*KO*^) showed developmental delay and die perinatally (19, 54). Thus, we first tested the viability of homozygous *Oga*^*D285A*^ animals by monitoring progeny of heterozygous breeding parents. No deviation from the expected Mendelian inheritance ratio of WT (25%), heterozygous (50%), and homozygous (25%) embryos at 15.5 and 18.5 dpc was observed, yet only a single homozygous animal (0.5% of 198 pups genotyped) survived to weaning stage (23–37 days after birth) (Fig. 2*A*). Homozygous *Oga*^*D285A*^ embryos at 18.5 dpc showed no obvious anatomical defects, although they appeared smaller than their littermates (Fig. 2*B*). The weight and volume of homozygous *Oga*^*D285A*^ 18.5 dpc embryos (weight: 1023 ± 175 mg, n = 15; volume: 887 ± 86 mm^3^, n = 8) were significantly reduced compared with WT (weight: 1197 ± 107.1 mg, n = 16; volume: 1020 ± 62 mm^3^, n = 8) and heterozygous embryos (weight: 1176 ± 124 mg, n = 50; volume: 1038 ± 88 mm^3^, n = 8) (Fig. 2, *C* and *D*). Taken together, these experiments show that catalytic deficiency of OGA leads to perinatal lethality and reduced growth.

### *O*-GlcNAc homeostasis is altered in *Oga*^*D285A*^ mice in a tissue-specific manner

**Figure 3:**
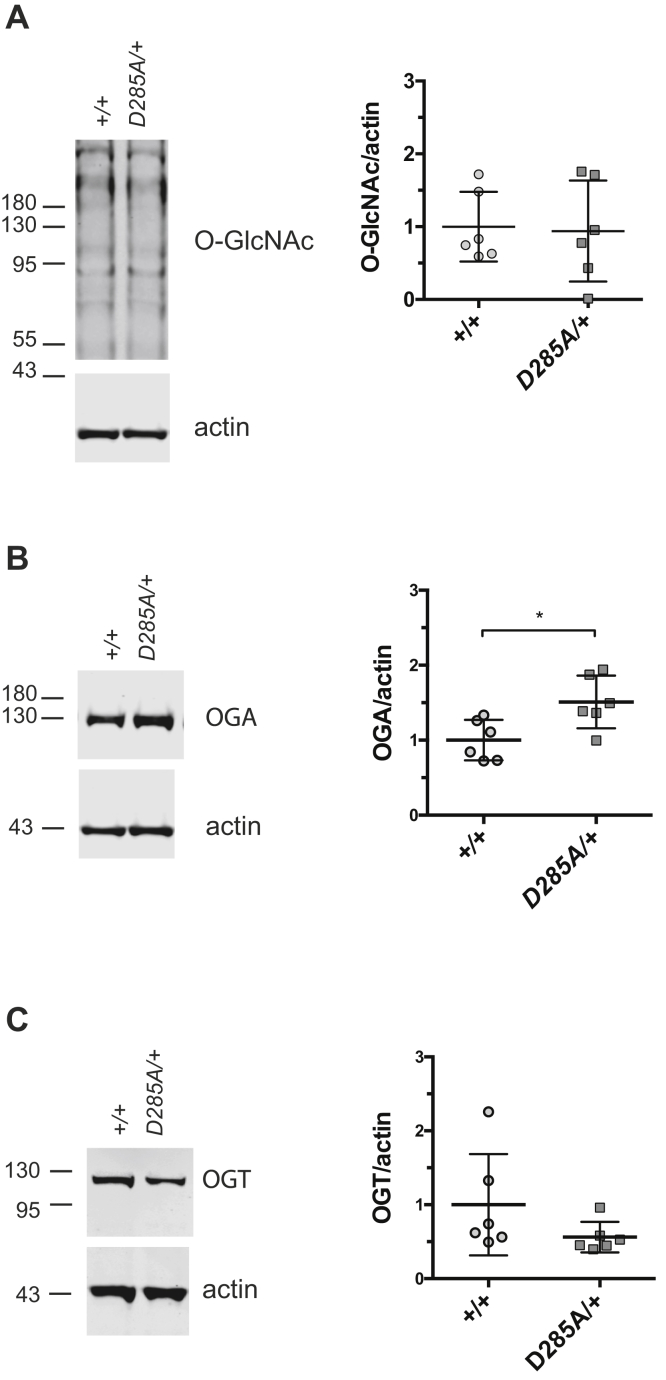
***O*-GlcNAc levels are maintained in adult *Oga***^***D285A/+***^**animals.** Data were analyzed using unpaired *t* test, n = 6 for all genotypes. *A*, Western blot of adult brain samples probed with the RL2 monoclonal antibody raised against *O*-GlcNAc–modified nucleoporins and actin antibodies. Actin was used as a loading control. Quantification of *O*-GlcNAcylated proteins revealing no difference in adult heterozygous *Oga*^*D285A/+*^ animals compared with WT control (*p* = 0.865). *B*, Western blot probed with anti-OGA and actin antibodies. Quantification of OGA protein levels indicated that adult heterozygous *Oga*^*D285A/+*^ animals show an increase of OGA protein levels compared with WT control (∗*p* = 0.018). *C*, Western blot probed with anti-OGT and actin antibodies. Quantification of OGT protein levels reflecting no difference in adult heterozygous *Oga*^*D285A/+*^ animals compared with WT control (*p* = 0.164). OGA, *O*-GlcNAcase; OGT, *O*-GlcNAc transferase.

Although homozygous *Oga*^*D285A*^ mice are not viable, the heterozygous *Oga*^*D285A/+*^ animals survived to adulthood. Previous studies have revealed increased protein *O*-GlcNAcylation in heterozygous *Oga*^*KO/+*^ hippocampus (29) and liver (54). We tested *O*-GlcNAc levels in the *Oga*^*D285A/+*^ mice to assess *O*-GlcNAc homeostasis. Mouse brain tissue obtained from 55- to 63-day-old WT and heterozygous *Oga*^*D285A/+*^ adult animals (n = 6 per group) were analyzed using an antibody specific for *O*-GlcNAcylated proteins. Protein *O*-GlcNAcylation was comparable in WT (mean ± SD fold change to WT, 1.1 ± 0.5 fold change) and heterozygous *Oga*^*D285A/+*^ samples (0.9 ± 0.7 fold change) (Fig. 3*A*). It has been established that OGT and OGA protein levels are sensitive to changes in protein *O*-GlcNAcylation in mammalian cells (34, 60). We next investigated such possible compensatory mechanisms by assessing OGA and OGT protein levels. Western blot revealed an increase of OGA protein level in heterozygous *Oga*^*D285A/+*^ animals (OGA: 1.5 ± 0.4 fold change) compared with WT animals (OGA: 1.0 ± 0.3 fold change). A decrease of OGT protein level in *Oga*^*D285A/+*^ (OGT: 0.6 ± 0.2 fold change) compared with WT (OGT: 1.0 ± 0.7 fold change) did not reach statistical significance (*t* test, *p* = 0.164). Our data indicate that heterozygous loss of OGA catalytic activity is compensated by altered OGA protein levels in adult heterozygous *Oga*^*D285A/+*^ animals to maintain normal level of protein *O*-GlcNAcylation (Fig. 3, *B* and *C*).

**Figure 4:**
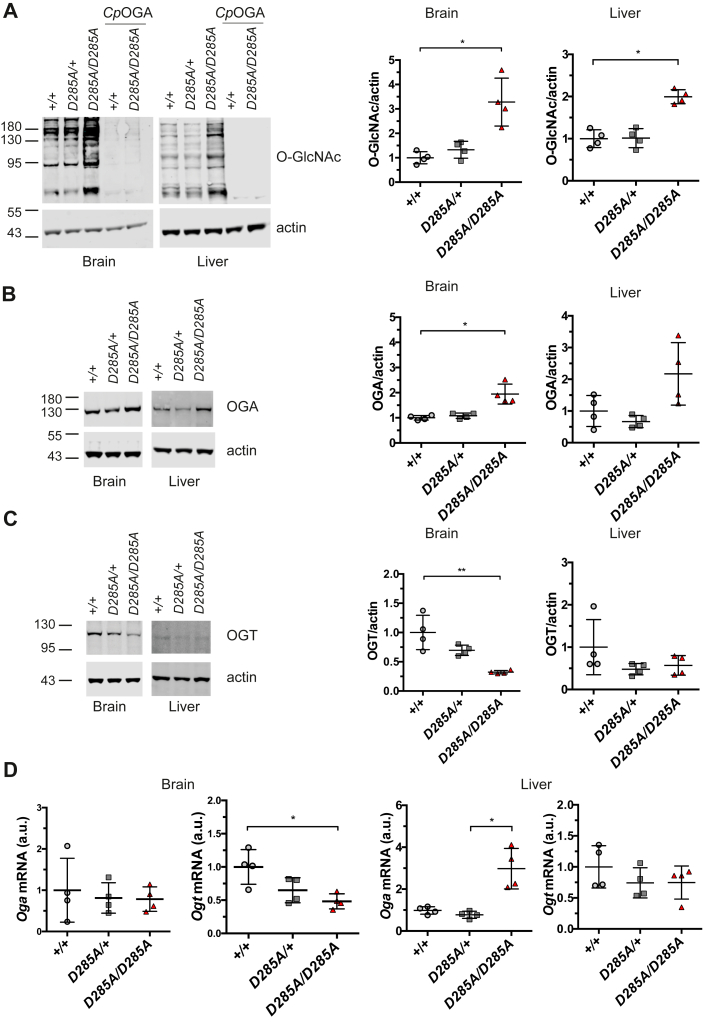
***O*-GlcNAc homeostasis is altered in 15.5 dpc *Oga***^***D285A/D285A***^**embryos.** Data were analyzed using one-way ANOVA with Kruskal–Wallis multiple comparisons test, n = 4 for all genotypes. *A*, western blot probed with the RL2 monoclonal antibody. The signal is specific to *O*-GlcNAc–modified proteins, as clostridium perfringens O-GlcNAcase treatment removed most of the signal. Quantification of *O*-GlcNAcylated proteins in brain and liver tissues revealing that homozygous *Oga*^*D285A/D285A*^ embryos show increased *O*-GlcNAc levels compared with WT samples in both tissues (brain: 3.3-fold increase, ∗*p* = 0.018; liver: twofold increase, ∗*p* = 0.043) and heterozygous *Oga*^*D285A/+*^ samples (brain: 2.5-fold, *p* = 0.15; liver: twofold, *p* = 0.073). *B*, western blot probed with anti-OGA and actin antibodies. Quantification of OGA protein levels in brain and liver tissues indicates that homozygous *Oga*^*D285A/D285A*^ samples have increased levels of OGA protein compared with WT control (brain: 1.9-fold increase, ∗*p* = 0.024; liver: 2.2-fold increase, *p* = 0.424) and heterozygous *Oga*^*D285A/+*^ samples (brain: 1.8-fold increase, *p* = 0.1186; liver: 3.3-fold increase, *p* = 0.056). *C*, western blot probed with anti-OGT and actin antibodies. Quantification of OGT protein levels in brain and tissues indicated that homozygous *Oga*^*D285A/D285A*^ samples show a decrease in OGT protein levels compared with WT control (brain: threefold reduction, ∗∗*p* = 0.01; liver: twofold reduction, *p* = 0.51) and heterozygous *Oga*^*D285A/+*^ samples (brain: 2.2-fold reduction, *p* = 0.233; liver: 1.8-fold reduction, *p* > 0.999). *D*, quantification of *Oga* mRNA levels indicated no differences for all genotypes in the brain, whereas in the liver, *Oga* mRNA levels are increased in homozygous *Oga*^*D285A/D285A*^ sample compared with WT control (threefold increase, *p* = 0.188) and heterozygous *Oga*^*D285A/+*^ samples (3.8-fold increase, ∗*p* = 0.013). Quantification of *Ogt* mRNA levels indicated that homozygous *Oga*^*D285A/D285A*^ (twofold reduction, ∗*p* = 0.033) and heterozygous *Oga*^*D285A/+*^ (1.5-fold reduction, *p* = 0.35) samples show a decrease in *Ogt* mRNA levels compared with WT control in the brain, whereas no differences in *Ogt* mRNA levels were observed between all genotypes in the liver. OGA, *O*-GlcNAcase; OGT, *O*-GlcNAc transferase.

Next, we investigated the effect of the D285A mutation on protein *O*-GlcNAcylation in homozygous *Oga*^*D285A*^ embryos. Previous mouse *Oga*^*KO*^ studies showed global elevation of *O*-GlcNAc level defects in brain and liver (54, 55). Thus, brain and liver samples isolated from *Oga*^*D285A*^ 15.5 dpc mouse embryos (n = 4 per group) were subjected to Western blotting. This revealed an increase of *O*-GlcNAcylation in brain tissues from homozygous *Oga*^*D285A/D285A*^ embryos (3.3 ± 1.0 fold change), whereas *O*-GlcNAcylation levels were similar in WT (1.0 ± 0.2 fold change) and heterozygous *Oga*^*D285A/+*^ samples (1.3 ± 0.3 fold change) (Fig. 4*A*). Similarly, an increase of *O*-GlcNAc levels was observed in liver samples from homozygous *Oga*^*D285A/D285A*^ embryos (2.0 ± 0.2 fold change), whereas *O*-GlcNAcylation was similar to WT level (1.0 ± 0.2 fold change) in heterozygous *Oga*^*D285A/+*^ liver samples (1.3 ± 0.2 fold change) (Fig. 4*A*). In the brain, a twofold increase of OGA protein levels and an approximately threefold reduction of OGT protein levels were detected in homozygous *Oga*^*D285A/D285A*^ samples (OGA: 1.9 ± 0.4, OGT: 0.3 ± 0.03 fold change) compared with WT brain samples (Fig. 4, *B* and *C*). OGT and OGA protein levels appeared similar in WT samples (OGA: 1.0 ± 0.1, OGT: 1.0 ± 0.3 fold change) and heterozygous *Oga*^*D285A/+*^ samples (OGA: 1.1 ± 0.1, OGT: 0.7 ± 0.1 fold change). Next, we investigated *Oga* and *Ogt* mRNA levels. No detectable differences in brain *Oga* mRNA levels were observed between the three genotypes (*Oga*^*+/+*^: 1.0 ± 0.8, *Oga*^*D285A/+*^: 0.8 ± 0.4, and *Oga*^*D285A/D285A*^: 0.8 ± 0.3 fold change) (Fig. 4*D*). However, reductions of *Ogt* mRNA levels were apparent in homozygous *Oga*^*D285A/D285A*^ (0.5 ± 0.1 fold change) and heterozygous *Oga*^*D285A/+*^ (0.6 ± 0.2 fold change) embryos, respectively, compared with WT samples (1.0 ± 0.3 fold change), although the differences between heterozygous and WT samples did not reach statistical significance (*p* = 0.35, Kruskal–Wallis multiple comparisons test) (Fig. 4*D*). In the embryo liver, an increase in OGA protein level was detected in homozygous *Oga*^*D285A/D285A*^ samples (OGA: 2.2 ± 0.98) compared with WT liver samples. Although a reduction in OGT protein level (OGT: 0.6 ± 0.2) was apparent, the difference did not reach statistical significance (*p* = 0.51, Kruskal–Wallis multiple comparisons test). No difference in OGA and OGT protein levels was observed between heterozygous (OGA: 0.7 ± 0.2, OGT: 0.5 ± 1.1) and WT embryos (OGA: 1.0 ± 0.5, OGT: 1.0 ± 0.7) (Fig. 4, *B* and *C*). An increase of *Oga* mRNA levels was apparent in homozygous *Oga*^*D285A/D285A*^ (3.0 ± 1.0) compared with WT samples (1.0 ± 0.2) and heterozygous *Oga*^*D285A/+*^ embryos (0.8 ± 0.2). No detectable differences in liver *Ogt* mRNA levels were observed between the three genotypes (*Oga*^*+/+*^: 1.0 ± 0.3, *Oga*^*D285A/+*^: 0.7 ± 0.2, and *Oga*^*D285A/D285A*^: 0.7 ± 0.3 fold change) (Fig. 4*D*). Taken together, these results suggest that loss of OGA catalytic activity leads to altered *O*-GlcNAc homeostasis in *Oga*^*D285A*^ mice.

### Microcomputed tomography reveals widespread organ defects in *Oga*^*D285A*^ embryos

**Figure 5:**
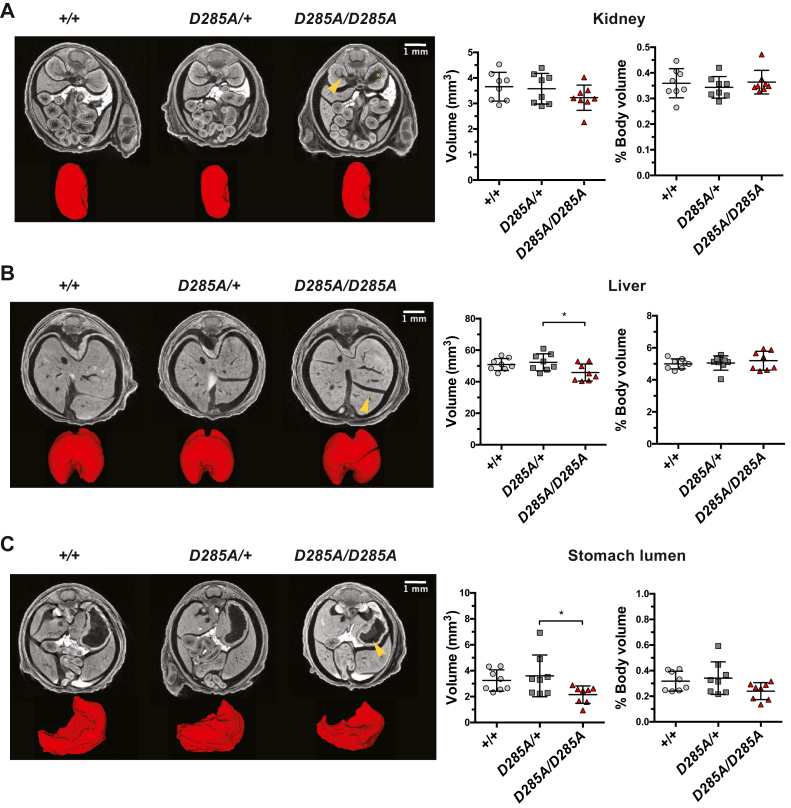
**Microcomputed tomography (microCT) reveals widespread developmental defects in 18.5 dpc *Oga***^***D285A***^**embryos.** Data were analyzed using one-way ANOVA with Tukey's multiple comparisons test, n = 8 for all genotypes. The scale bars for the grayscale sections are represented. *A*, representative microCT images of axial sections of the abdomen region and 3D volume renderings of the right kidney (in *red*) from a WT, heterozygous *Oga*^*D285A/+*^, and homozygous *Oga*^*D285A/D285A*^ 18.5 dpc embryos. The renal pelvis appeared dilated in the *Oga*^*D285A/D285A*^ embryos (*yellow arrow*) and is associated with advanced unilateral hydronephrosis in two animals (*yellow asterisk*). Quantification of the volume of the right kidney showed no difference in kidney volume between all genotypes after normalization to whole embryo body. *B*, representative microCT images of axial sections and 3D volume renderings (in *red*) of the liver from a WT, heterozygous *Oga*^*D285A/+*^, and homozygous *Oga*^*D285A/D285A*^ 18.5 dpc embryos. The intralobular spaces from the homozygous *Oga*^*D285A/D285A*^ appeared enlarged as shown with *yellow arrow* compared with WT control. Quantification of the volume of the liver showed no difference in liver size between all genotypes after normalization to whole embryo body. *C*, representative microCT images of axial sections of the abdomen region and 3D volume renderings of the stomach lumen (in *red*) from WT, heterozygous *Oga*^*D285A/+*^, and homozygous *Oga*^*D285A/D285A*^ 18.5 dpc embryos. The stomach lumen from the homozygous *Oga*^*D285A/D285A*^ mice appeared reduced as shown with *yellow arrow* compared with WT and heterozygous *Oga*^*D285A/+*^ embryos. Quantification of the volume of the stomach lumen showed a possible reduced size of the lumen in homozygous *Oga*^*D285A/D285A*^ compared with heterozygous *Oga*^*D285A/+*^ (*p* = 0.102) and WT (*p* = 0.243) embryos after normalization to whole embryo body although this did not reach statistical significance.

**Figure 6:**
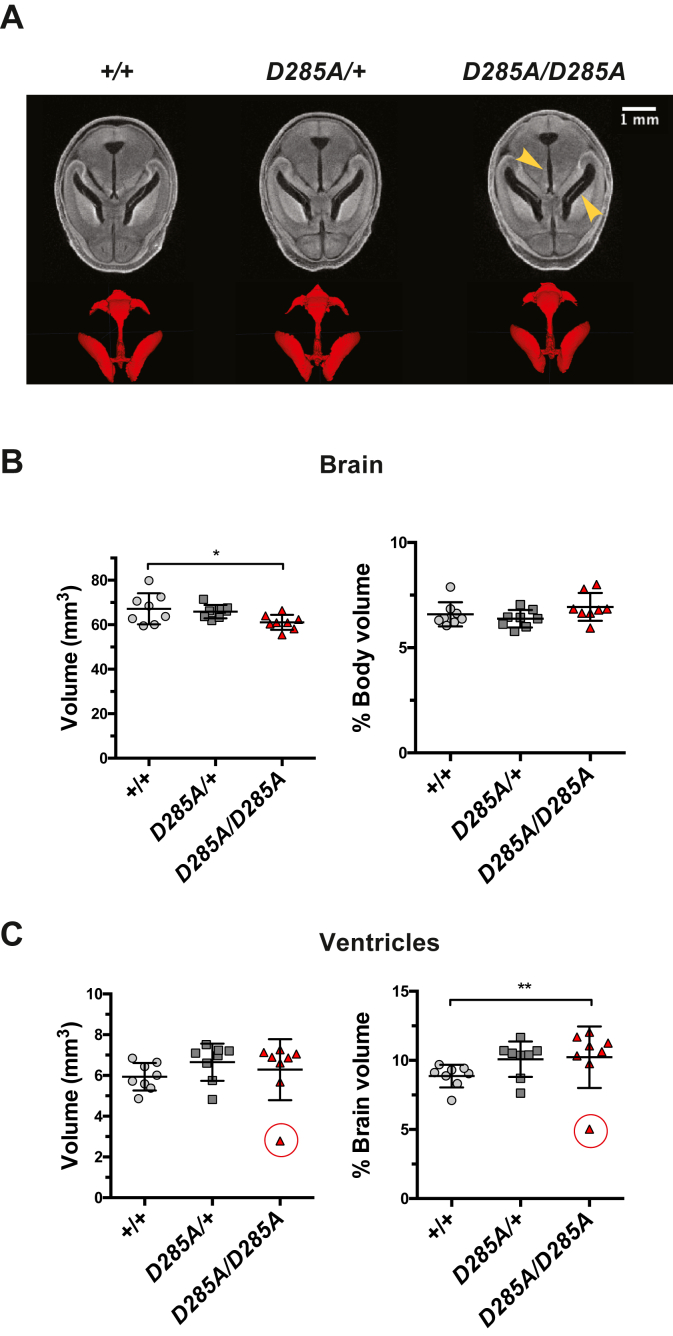
***Oga***^***D285A/D285A***^**embryos show enlarged brain ventricles.** Data were analyzed using one-way ANOVA with Tukey's multiple comparisons test, n = 8 for all genotypes. The scale bars for the grayscale sections are represented. *A*, representative microcomputed tomography images of axial sections of the brain and 3D volume renderings of the ventricular system from a WT, heterozygous *Oga*^*D285A/+*^, and homozygous *Oga*^*D285A/D285A*^ 18.5 dpc embryos. *B*, quantification of the volume of the brain showed no differences between all embryo genotypes after normalization to whole embryo body. *C*, quantification of the volume of the brain ventricles showing possible increases of the ventricular system in heterozygous *Oga*^*D285A/+*^ (*p* = 0.281) and homozygous *Oga*^*D285A/D285A*^ embryos (*p* = 0.210) compared with WT control embryos after normalization to brain volume. A Dixon's Q test identified the smallest value (*circled*) in homozygous *Oga*^*D285A/D285A*^ group as an outlier. Once this value is excluded from the analysis, the difference between *Oga*^*D285A/D285A*^ and the WT control becomes statistically significant (∗∗*p* = 0.002) using one-way ANOVA with Tukey's multiple comparisons test.

We next performed an unbiased in-depth analysis of growth and morphology on 18.5 dpc mouse embryos to identify organ defects that could explain perinatal lethality specific to loss of OGA catalytic activity. We employed X-ray microcomputed tomography (microCT) to capture the anatomy of intact whole embryos. In total, 70 anatomical structures were scored in WT, heterozygous, and homozygous embryos (n = 8 per group). Analysis of microCT imaging revealed anatomical abnormalities in 26 regions across all genotypes (Table S1). Two WT embryos showed a single abnormality, one in the brain and one in the heart. Three heterozygous *Oga*^*D285A/+*^ embryos exhibited morphological defects in eight regions, including heart, stomach, and kidney (Table S1). The majority of the abnormalities were observed in homozygous *Oga*^*D285A/D285A*^ embryos with seven samples revealing defects in 21 areas (Table 1). For these regions, all WT *Oga*^*+/+*^ embryos appeared normal, whereas morphology of the stomach lumen (two cases) and kidney (one case) was affected in heterozygous *Oga*^*D285A/+*^ embryos. We decided to focus on essential organs that were affected in at least four *Oga*^*D285A/D285A*^ samples, namely the kidneys, liver, stomach, and brain ventricles. Half of the embryos showed abnormalities in the kidneys exhibiting dilated renal pelvis that developed to advanced unilateral hydronephrosis in two *Oga*^*D285A/D285A*^ embryos (Fig. 5*A*). Enlarged intralobular space in the liver was observed in 75% of *Oga*^*D285A/D285A*^ animals, whereas in the two most severe cases, it was associated with reduced left/right and caudal lobes (Fig. 5*B*). The lumina of the stomach appeared reduced in half of the *Oga*^*D285A/D285A*^ embryos (Fig. 5*C*). In addition, there were abnormalities in the fourth brain ventricles in five cases suggesting developmental defects in the brain (Fig. 6*A*).

**Table 1:** List of abnormalities found in heterozygous *Oga*^*D285A/+*^ and homozygous *Oga*^*D285A/D285A*^ embryos

The number of affected embryos (n = 8 per genotype) for each abnormality is reported. *Green* indicates no defects found, and *yellow–red* gradient indicates that abnormal features were detected.

To explore possible volume differences in the affected organs, 3D segmentation was performed. We detected a reduction of volume for the liver, kidney, stomach lumen, and brain in the *Oga*^*D285A/D285A*^ animals and an increase of volume in brain ventricles (Figs. 5 and 6). To compensate for the reduced body size of the *Oga*^*D285A/D285A*^ animals, the volumes of these organs were then normalized to the volume of the whole embryo or to the volume of the brain in case of ventricle size analysis. No difference in kidney volume normalized to the embryo's whole-body volume was observed between genotypes (*Oga*^*+/+*^: 0.36 ± 0.06%, *Oga*^*D285A/+*^: 0.34 ± 0.04%, and *Oga*^*D285A/D285A*^: 0.36 ± 0.05% of whole-body volume) (Fig. 5*A*). Despite the apparent enlarged interlobular space, the relative size of the liver was not affected in *Oga*^*D285A/D285A*^ mice (*Oga*^*+/+*^: 5.0 ± 0.3%, *Oga*^*D285A/+*^: 5.1 ± 0.4%, and *Oga*^*D285A/D285A*^: 5.2 ± 0.6% of whole-body volume) (Fig. 5*B*). However, a possible reduction in stomach lumina volume was observed in *Oga*^*D285A/D285A*^ mice (*Oga*^*+/+*^: 0.32 ± 0.08%, *Oga*^*D285A/+*^: 0.34 ± 0.13%, and *Oga*^*D285A/D285A*^: 0.24 ± 0.07% of whole-body volume), although this did not reach statistical significance (*Oga*^*+/+*^ *versus Oga*^*D285A/D285A*^: *p* = 0.243) (Fig. 5*C*). Similarly, no difference in normalized brain volume was observed between genotypes (*Oga*^*+/+*^: 6.6 ± 0.6%, *Oga*^*D285A/+*^: 6.4 ± 0.4%, and *Oga*^*D285A/D285A*^: 6.9 ± 0.7% of whole-body volume) (Fig. 6*B*). These volumetric measurements suggested that the gross development of the liver, kidneys, stomach, and brain was proportional to the development of the whole in *Oga*^*D285A/D285A*^ embryos. Next, we measured the volume of the brain ventricles, where a mild increase in *Oga*^*D285A/+*^ and *Oga*^*D285A/D285A*^ embryos compared with WT animals was detected (*Oga*^*+/+*^: 8.8 ± 0.8%, *Oga*^*D285A/+*^: 9.8 ± 1.2%, and *Oga*^*D285A/D285A*^: 10.01 ± 2.4% of whole-brain volume), although the difference did not reach statistical significance (*Oga*^*+/+*^ *versus Oga*^*D285A/+*^: *p* = 0.281, *Oga*^*+/+*^ *versus Oga*^*D285A/D285A*^: *p* = 0.210) (Fig. 6*C*). In the *Oga*^*D285A/D285A*^ group, one embryo showed very low ventricle volumes (circled value in Fig. 6*C*). This animal (identified as HOM1) appeared very small with a 24.1% reduction of body volume compared with the other *Oga*^*D285A/D285A*^ embryos, and it displayed the most severe phenotypes within 16 anatomical regions (Table S1). This embryo also exhibited an overall underdeveloped state with reduced volume of the kidney, stomach, and brain. A Dixon's Q test on the ventricle unnormalized volume value of this embryo indicated that this data point may be considered as an outlier. Therefore, we performed the analysis on the data set where this embryo was excluded. Interestingly, the analysis indicated an enlargement of the brain ventricles in *Oga*^*D285A/D285A*^ animals compared with WT animals with a difference reaching statistical significance (*Oga*^*+/+*^ *versus Oga*^*D285A/D285A*^: *p* = 0.002) suggesting abnormal neurodevelopment in *Oga*^*D285A/D285A*^ embryos. Taken together, these results reveal widespread organ defects albeit with incomplete penetrance arisen in *Oga*^*D285A/D285A*^ embryos as a consequence of catalytic deficiency of OGA.

## Discussion

Previous studies have established that *Oga* is essential for mammalian development (19, 54). However, it is not known whether this is linked to OGA activity or functions of the other OGA domains. Similar to the *Oga*^*KO*^ mouse models, the loss of OGA catalytic activity leads to reduced growth and perinatal lethality in our OGA catalytic-deficient model, suggesting that it is the loss of OGA enzymatic function that causes lethality and alteration in mouse embryonic development. In the *Oga*^*KO*^ models, defects in lungs and liver tissues were observed in the homozygous embryos using classic histological techniques. Here, we used microCT to investigate whether other organ defects could arise from loss of OGA activity. We showed that *Oga*^*D285A/D285A*^ pups, from the same mouse strain used for the *Oga*^*KO*^ models, display various organ-specific defects with varying penetrance among the embryos that were not previously described. The most prominent abnormalities were found in the kidney exhibiting dilatation of the renal pelvis and advanced hydronephritis, the latter is associated with kidney infection and kidney failure (61) posing a significant risk of postnatal death (62). Defects in the liver, stomach, and brain were observed in parallel, and accumulation of abnormalities in several organs could contribute to perinatal lethality in the *Oga*^*D285A/D285A*^ mice.

Loss of OGA activity resulted in increased global *O*-GlcNAc levels in homozygous *Oga*^*D285A/D285A*^ embryos supporting that the D285A mutation impairs OGA activity in mouse. Increased protein *O*-GlcNAcylation has been associated with several chronic pathologies, including cancers, osteoarthritis, diabetes, diabetic vascular dysfunction, and diabetic nephropathy (63, 64, 65, 66, 67). Hyperglycemia is a hallmark of diabetes, and high glucose levels induce elevation of global *O*-GlcNAcylation by increased flux through the hexosamine biosynthetic pathway. Raised *O*-GlcNAc levels were found in tissues from diabetic animals and humans (68, 69, 70) as well as in pups of diabetic female mice (71). Several studies showed that *O*-GlcNAcylation contributes to hyperglycemia-induced tissue damage in heart and kidney of adult patients (69). Furthermore, maternal hyperglycemia causes developmental delay associated with congenital defects, also called as diabetic embryopathy, affecting the kidneys, central nervous system, heart, and skeletal system among others (72, 73). Inhibition of OGT blocked the negative impact of hyperglycemia or glucosamine supplementation on blastocyst formation, cell number, and apoptosis during mouse embryogenesis (74). Reduction of global *O*-GlcNAcylation through OGT inhibition in diabetic pregnant mice *in vivo* reduced neural tube defects in embryos (75). In our *Oga*^*D285A*^ model, elevated *O*-GlcNAc levels independent of hyperglycemia were also associated with anomalies during mouse development, demonstrating a direct link between excess of *O*-GlcNAcylation and developmental defects.

Interestingly, we observed mild enlargement in the brain ventricles of both *Oga*^*D285A/+*^ and *Oga*^*D285A/D285A*^ embryos indicating that the phenotypes observed previously in brain-specific *Oga*^*KO*^ mice (55) are caused by the loss of catalytic activity of OGA. Ventricle enlargement, frequently associated with neurodevelopmental conditions including ID, originates from defects in neurogenesis, proliferation, or ciliary development (76, 77, 78). In the brain-specific *Oga*^*KO*^ model, an imbalance between neuronal proliferation and disturbed neurogenesis was detected in neonates (55) that can be linked to hippocampal-dependent spatial learning and memory defects observed in adult heterozygous *Oga*^*KO/+−*^. A common hallmark in OGT-CDG mutations associated with ID is the reduction in OGA expression, suggesting a possible mechanism that might contribute in part to the pathogenesis of ID (30, 32, 33, 34, 35). Our model could be used to further examine the role of OGA and its activity in neurodevelopment and OGT-CDG pathogenesis.

Loss of OGA activity resulted in increased global *O*-GlcNAc levels accompanied with an increase in OGA protein levels and a decrease in OGT protein levels, providing further evidence for a compensatory molecular response to maintain *O*-GlcNAc homeostasis in the brain in both *Oga*^*D285A/+*^ adults and *Oga*^*D285A/D285A*^ embryos. Although similar upregulation was observed for *O*-GlcNAc and OGA levels in the liver of embryos, OGT expression remained unchanged suggesting a tissue-specific response. Previous reports have shown that pharmacological inhibition of OGA activity led to an increase of *Oga* mRNA expression and OGA protein levels (60, 79, 80). Similarly, we detected increased *Oga* mRNA and OGA protein levels in liver samples from *Oga*^*D285A/D285A*^ embryos. The primer set used to detect *Oga* mRNA levels flank exon 1, allowing the evaluation of mature *Oga* mRNA levels. The data suggest that transcriptional upregulation of *Oga* mRNA levels occurs to produce more proteins to compensate for loss of catalytic activity. However, the levels of *Oga* mRNA remained unchanged in the brain of *Oga*^*D285A/D285A*^ embryos suggesting that OGA regulation in this tissue may occur at the post-transcriptional level. Interestingly, OGA is itself *O*-GlcNAcylated, which could provide a mechanism for such post-transcriptional regulation (81, 82).

The levels of OGT protein were reduced in the brain. Similarly, OGT protein levels were found decreased in association with reduction of OGA protein expression in models of two OGT-CDG mutations (32, 33). We showed that reduction of OGT protein levels was also associated with a reduction of *Ogt* mRNA levels. Taken together, these data indicate possible transcriptional control of OGT expression influenced by OGA activity. OGA-mediated regulation of *Ogt* transcription through cooperation with the HAT p300 and transcription factor CCAAT/enhancer-binding protein β has been described *in vitro* in WT primary mouse hepatocytes (83). In addition, elevated *O*-GlcNAc because of pharmacological inhibition of OGA promotes the retention of *Ogt* RNA intron 4 through the *Ogt* intronic splicing silencer leading to *Ogt* mRNA degradation (84). The primer set we used to evaluate the levels of *Ogt* mRNA levels flank exons 4/5, allowing the detection of the mature form of *Ogt* mRNA (85). Further experiments will be needed to evaluate whether the reduction of mature *Ogt* mRNA levels we observed in the brain is due to less *Ogt* mRNA produced or because of an increase in intronic retention leading to *Ogt* mRNA degradation. Together with our data, these studies suggest that loss of OGA activity induces alteration of *Ogt* transcription to maintain *in vivo O*-GlcNAc homeostasis in the brain but not in the liver during gestation. Although the precise mechanisms associated with OGA/OGT regulation in the *Oga*^*D285A*^ mice remain to be explored, our results indicate that mechanisms for *O*-GlcNAc homeostasis maintenance may differ from one tissue to another or only occur in some tissues. A similar strategy could be used to generate a mouse model carrying an inducible *Oga*^*D285A*^ allele to investigate the physiological consequences of prolonged OGA inhibition during adulthood.

A number of studies suggest that elevation of *O*-GlcNAcylation through pharmacological inhibition of OGA could provide therapeutic benefit for chronic neurological conditions, such as temporal lobe epilepsy (86), AD (44, 45, 46, 47), and amyotrophic lateral sclerosis (87). The degree of OGA inhibition needed to achieve the desired biological response while minimizing any potential side effects is an important consideration. In the brain, at least 80% of OGA inhibition is necessary to achieve global protein *O*-GlcNAcylation elevation *in vivo* (44). This is in accordance with our results showing that heterozygous *Oga*^*D285A/+*^ mice displayed unchanged global *O*-GlcNAc levels. Although heterozygous *Oga*^*D285A/+*^ mice are viable and do not develop overt developmental phenotypes, complete loss of OGA activity in homozygous *Oga*^*D285A/D285A*^ mice led to elevated *O*-GlcNAc levels, perinatal lethality, and abnormal development. The phenotypes observed in the *Oga*^*D285A/D285A*^ mice suggest the possibility of adverse on-target side effects upon prolonged inhibition of OGA. Although the use of adult and embryo tissues is a different context and no apparent toxic effects have been reported to date because of prolonged exposure of OGA inhibitors in adult animals, the investigations are mainly limited to the brain (44, 47, 88), and the effects of long-term OGA inhibition in other tissues remain unclear.

Two splice variants of human OGA exist: (1) a full-length nucleocytoplasmic isoform that contains the N-terminal glycoside hydrolase domain together with the C-terminal HAT domain and (2) a short nuclear isoform that lacks the HAT domain and shows lower OGA activity compared with the full-length isoform (89, 90). OGA undergoes caspase-3 cleavage giving rise to two fragments that individually lack OGA catalytic activity. However, the two fragments can reassemble and restore fully functional OGA in cells (51). This suggests that the C-terminal part of OGA that includes the HAT domain may be important for catalytic activity. Although it remains unknown whether this domain possesses any additional functions, phenotypic comparison of *Oga*^*D285A*^ and *Oga*^*KO*^ mouse models reveals that the OGA domain is essential for late embryonic development and perinatal survival.

In summary, we generated a mouse model lacking OGA catalytic activity, providing a genetic model for prolonged inhibition of OGA *in vivo.* This model will be useful to study the role of *O*-GlcNAcylation in development and to understand the potential function of the noncatalytic OGA domains. We showed that loss of OGA activity leads to perinatal lethality, organ defects, and tissue-specific disruption in *O*-GlcNAcylation homeostasis in mice.

## Experimental procedures

### Generation of *Oga*^*D285A*^ knock-in mice

*Oga*^*D285A*^ knock-in (C57BL/6NTac-*Mgea5*^*tm3592(D285A)Arte*^) mice were generated by Taconic Artemis GmbH *via* insertion of the constitutive knock-in allele and subsequent Flpe-mediated deletion of a puromycin resistance cassette (Fig. 1*C*). First, *Oga*^*D285A*^ mESCs were created *via* insertion of the knock-in allele *via* homologous recombination in C57BL/6NTac (Art B6 3.6) mESCs. The targeting vector coding for a ∼10-kb-sequence spanning exon 4 to 8 of the *Oga* gene was electroporated into mESCs. The construct also contained a flippase recognition target–flanked puromycin resistance cassette in the intronic region between intron 6 and 7 allowing for subsequent isolation of recombinant clones (Fig. 1*C*). Correct homologous recombination and single integration at both 5’ and 3’ sides in mESCs clones was validated using the cag probe that detects a region located within the puromycin selection cassette. The genomic DNA was digested with either *Sph*I for the 5’ side or *Eco*RV for the 3’ side and analyzed by Southern blot. The presence of the D285A mutation and the single integration was determined by PCR using 10328_1 and 10328_2 primers followed by sequencing using the 10610_135 primer.

Then, 10 to 15 cells from three selected mESC colonies bearing the D285A mutation were injected into 3.5-days blastocysts from BALB/c female mice (BALB/cAnNTac; Taconic Artemis GmbH) in Dulbecco's modified Eagle's medium supplemented with 15% fetal calf serum under mineral oil. After recovery, 48 to 51 blastocysts were transplanted into nine pseudopregnant NMRI females (BomTac:NMRI; Taconic Artemis GmbH). Ten highly chimeric progenies (>50%) were selected based on their coat color and bred with flippase (Flp) deleter C57BL/6 mice (C57BL/6-Tg(CAG-Flpe)2Arte; Taconic Artemis GmbH) to eliminate the puromycin cassette. Eight pups heterozygous for the D285A mutation were used as colony founders. Primers used for genotyping and validation are listed in Table S2.

### Animal husbandry and genotyping

Founder *Oga*^*D285A/+*^ heterozygous mice were crossed to C57BL/6J WT animals (Charles River UK Limited) inhouse. The line was initially bred by intercrossing heterozygous animals, then maintained by backcrossing to C57BL/6J background for two generations. Animals were housed in standard holding cages with water and food available *ad libitum* and 12/12 h light/dark cycles throughout the study. All animal studies and breeding were performed in accordance with the Animal (Scientific Procedures) Act of 1986 for the care and use of laboratory animals. Procedures were carried under United Kingdom Home Office Regulation with approval by the Welfare and Ethical Use of Animals Committee of University of Dundee.

Genotyping was performed by diagnostic PCR using *Thermococcus kodakaraensis* Hot Start DNA polymerase (EMD Millipore) on genomic DNA isolated from ear-notch biopsy with 10609_129 and 10609_130 primers (Table S2) that amplified 395 base pair fragment of the knock-in and 320 base pair fragment of the WT allele. Animals were genotyped after weaning at 23 to 37 days old.

### Weight measurement

Weights of the embryos were determined after fixation on analytical scale. Excess liquid from the surface of the embryos was removed with paper towels prior to measurement. Statistical significance was calculated using one-way ANOVA with Tukey's post hoc test.

### Western blotting

Tissues were rapidly dissected, rinsed in cold PBS, snap frozen in liquid nitrogen, and stored at −80 °C. For immunoblotting, tissue was lysed in 50 mM Tris–HCl (pH 7.4), 0.1 mM EGTA, 1 mM EDTA, 1% Triton X-100, 1 mM sodium orthovanadate, 50 mM sodium fluoride, 5 mM sodium pyrophosphate, 0.27 M sucrose, 0.1% 2-mercaptoethanol supplemented with protease inhibitors (1 mM benzamidine, 0.2 mM PMSF, and 5 μM leupeptin), and 10 μM GlcNAcstatin G. Lysates were centrifuged at 14,000 rpm for 20 min at 4 °C, and the protein concentration was determined with Pierce 660 nm protein assay (Thermo Scientific). About 20 to 30 μg of protein was denatured in SDS loading buffer containing 1% 2-mercaptoethanol. Proteins were separated on precast 4 to 12% NuPAGE Bis–Tris Acrylamide gels (Invitrogen) and transferred to nitrocellulose membrane. Membranes were incubated with primary antibodies in blocking buffer and 5% bovine serum albumin in Tris-buffered saline with 0.1% Tween-20 overnight at 4 °C. Anti-OGA (1:500 dilution; catalog no. HPA036141; Sigma), anti-*O*-GlcNAc (RL2) (1:500 dilution; NB300-524, Lot# A-2; Novus Biologicals), anti-OGT (F-12) (1:1000 dilution; sc-74546; Santa Cruz), and antiactin (1:2000 dilution; A2066; Sigma) antibodies were used. Next, the membranes were incubated with IR680/800-labeled secondary antibodies (Licor) at room temperature for 1 h. Blots were imaged using a Li-Cor Odyssey infrared imaging system (Li-Cor), and signals were quantified using Image Studio Lite software (Licor). Results were normalized to the mean of each corresponding WT replicates set and represented as a fold change relative to WT. Significance was calculated using one-way ANOVA with Tukey's multiple comparisons test or Student's *t* test.

### C*p*OGA treatment

Mouse embryo brain and liver lysates lacking GlcNAcstatin G were treated with recombinant clostridium perfringens O-GlcNAcase (*Cp*OGA) to test the specificity of the anti-*O*-GlcNAc antibody (RL2). Recombinant glutathione-*S*-transferase–tagged *Cp*OGA was expressed and purified as described earlier (91). Lysates containing 60 μg protein at 0.85 μg/ml concentration were incubated with 10 μg recombinant WT CpOGA for 60 min at 30 °C. The reaction was stopped by addition of SDS loading buffer containing 1% 2-mercaptoethanol and boiling the samples for 5 min.

### Real-time quantitative PCR

Total RNA was purified using RNAeasy Kit (Qiagen), and then 1000 ng of sample RNA was used for reverse transcription with the qScript cDNA Synthesis Kit (Quantabio). Quantitative PCR reactions were performed using the PerfeCTa SYBR Green FastMix for iQ (Quantabio) reagent, in the CFX Connect Real-Time PCR Detection System (BioRad), employing a thermocycle of one cycle at 95 °C for 30 s and then 40 cycles at 95 °C for 5 s, 60 °C for 15 s, and 68 °C for 10 s. Data analysis was performed using CFX Manager software (BioRad). Samples were assayed in biological quadruplicates with technical triplicates using the comparative Ct method. The threshold-crossing value was normalized to internal control transcripts (*Gapdh*, *Actb*, and *Pgk1*). Primers used are listed in Table S3. Results were normalized to the mean of each corresponding WT replicates set and represented as a fold change relative to WT. Statistical significance was evaluated using one-way ANOVA with Kruskal–Wallis multiple comparisons test on ΔCt values obtained for each biological replicate.

### Sample preparation and imaging parameters for X-ray microCT

Crosses between heterozygous *Oga*^*D285A/+*^ female and *Oga*^*D285A/+*^ male mice were set up, and plug formation in the females was monitored to determine day 0.5 after fertilization. Pregnant females were sacrificed 18 days after plug formation with increasing concentration of carbon dioxide, and the femoral artery was severed. Embryos of 18.5 dpc were dissected into 6-well plate containing ice-cold PBS (pH 8). Embryos were washed twice with ice-cold PBS, and small tail samples were collected for genotyping. Embryos were fixed in ice-cold 4% paraformaldehyde in phosphate buffer solution for 7 days on roller at 4 °C. Fixed embryos were stored in 1% paraformaldehyde in phosphate buffer until shipment and further processing. At MRC Harwell, samples were rinsed in distilled water, then placed in ∼15 ml 50% Lugol's solution prepared in MilliQ distilled water, and kept at room temperature on the rocker, in vials wrapped in tinfoil. About 50% Lugol's solution was changed every other day for 14 days to achieve contrasting. Embryos were rinsed with MilliQ distilled water for a minimum of 1 h before they were embedded in 1% w/v Iberose-High Specification Agarose (AGR-500) made in MilliQ distilled water and left at room temperature to set for 2 h. 3D imaging of samples was performed using a Skyscan 1172 microCT scanner (Bruker) with aluminium filter setting, scanning with 70 kV, the image pixel size set to 5 μm, employing a rotation step 0.25° while achieving 180° rotation along the anterior–posterior axis.

### Image processing

Acquired images were reconstructed using NRecon Reconstruction (Bruker) software. Data were further cropped, and TIFF stacks were generated using Harwell Automated Recon Processor (HARP; Harwell) software. Image series were converted into .nrrd file format and viewed using open-source Fiji-based 3D Viewer and on 3D Slicer (version 4.10.1; https://www.slicer.org/), an open-source medical image processing and visualization system. Segmentation and 3D volume rendering for detailed morphological analysis were performed with the ITK-SNAP medical image segmentation tool (version 3.6.0 (92)). To eliminate bias, image stacks were renamed, and the genotype was blinded for the analysis. Statistical significance was calculated using one-way ANOVA with Tukey's multiple comparisons test. The Dixon's Q test was performed for outlier identification in the sample groups according to Rorabacher (93). The experimental Q value (Q_exp_) was calculated from raw data using Q_exp_ = (x_2_ − x_1_)/(x_n_ − x_1_), where x_1_ is the value of interest, x_2_, the closest value to x_1_ in the data set, and x_n_ the highest value in the data set. Q_exp_ was next compared with the Q critical value (Q_crit_) of 0.526 for n = 8 for a confidence level of 95% using the Q-table. Data that displayed a Q_exp_ higher than the Q_crit_ of 0.526 were considered as an outlier at a confidence level of 95%.

## Data availability

All data are contained in the article.

## Supporting information

This article contains supporting information.

## Conflict of interest

The authors declare that they have no conflicts of interest with the contents of this article.
