# Supplementary material for: Loss of O-GlcNAcase catalytic activity leads to defects in mouse embryogenesis
Source: J Biol Chem. 2021 Feb 19;296:100439. doi: 10.1016/j.jbc.2021.100439 (PMC7988489; doi:10.1016/j.jbc.2021.100439)
Supplement: Supplemental Tables S1–S3 [file mmc1.pdf]

Supplementary figure legends

Supplementary Table S1. List of abnormalities found in *Oga*$^{+/+}$, *Oga*$^{D285A/+}$ and *Oga*$^{D285A/D285A}$ embryos.

Supplementary Table S2. Primers used for genotyping the *Oga*^{D285A} mouse line.

Supplementary Table S3. Primers used for qPCR analysis.

	Sample ID	Abnormalities found
Oga$^{+/+}$	WT 1	
	WT 2	
	WT 3	
	WT 4	
	WT 5	
	WT 6	
	WT 7	Interventricular septum
	WT 8	Midbrain roof
Oga$^{D285N/+}$	HET 1	Kidney
	HET 2	
	HET 3	
	HET 4	
	HET 5	
	HET 6	
	HET 7	Stomach: Pyloric antrim, Stomach: Fundus, Stomach: Lumen, Ventricles, Tricuspid valve, Mitral valve, Interventricular septum
	HET 8	Stomach: Pyloric antrim, Stomach: Fundus, Stomach: Lumen
Oga^{D285N}	HOM 1	Trachea, Vibrissae, Main bronchus, Lateral ventricles, 4th ventricle, Emerging cervical spinal cord, Intralobular space, Stomach: Pyloric antrim, Stomach: Fundus, Stomach: Lumen, Intra-retinal space, Hyaloid cavity, Eustachian tube, Lower molar teeth, Spleen, Upper molar teeth
	HOM 2	Vibrissae, Main bronchus, 4th ventricle, 3rd ventricle, Intralobular space, Stomach: Lumen, Intra-retinal space, Kidney, Spleen
	HOM 3	Vibrissae, Main bronchus, 4th ventricle, 3rd ventricle, Intralobular space, Stomach: Lumen, Intra-retinal space
	HOM 4	4th ventricle, Liver: Caudate lobe, Intralobular space, Stomach: Lumen, Intra-retinal space, Kidney
	HOM 5	Intralobular space, Kidney
	HOM 6	4th ventricle, Kidney
	HOM 7	Liver: R/L lobes, Liver: Caudate lobe, Intralobular space, Bladder
	HOM 8	

Supplementary Table S1. List of abnormalities found in *Oga*$^{+/+}$, *Oga*$^{D285N/+}$ and *Oga*^{D285N} embryos.

Application	Name	Sequence
Amplification of DNA region harbouring the D285A mutation for sequencing in mES cells	10328_1	TATGGGAACATACGTCATTATTGACG
	10328_2	GACACTACAGATACCTGCATGTCTTCC
Amplification of DNA region harbouring the D285A mutation for sequencing after Flpe-mediated deletion of the puromycin resistance cassette	10610_135	TTAAGGATGCTTAGGAGCTGC
	10610_136	CTTGTACCAAGTGGCAAGGG
Detection of puromycin cassette	6547_1	AAGCGGGCATGACTTCTGC
	6547_2	GGGCTTGTACTCGGTCATGG
Detection the Flpe transgene	Flpe_as_	GGCAGAAGCACGCTTATCG
	Flpe_s_	GACAAGCGTTAGTAGGCACAT
Routine genotyping of *Oga*^{D285A} mice	10609_129	TGTACATCACATGCCAGAAGAGG
	10609_130	TGCACATATATGCACGCAGG

Supplementary Table S2. Primers used for genotyping the *Oga*^{D285A} mouse line.

List of Primers	Sequence (5'->3')
Actb Forward	GATCAAGATCATTGCTCCTCCTG
Actb Reverse	CAGCTCAGTAACAGTCCGCC
Gapdh Forward	ACCCTTAAGAGGGATGCTGC
Gapdh Reverse	GGGACGAGGAAACACTCTCC
Pgk1 Forward	GCTATCTTGGGAGGCGCTAA
Pgk1 Reverse	AAAGGCCATTCCACCACCAA
Ogt Forward	CTGTGTTCGCAGTGACCT
Ogt Reverse	CAGCCAAATCTCCCCTTGTG
Oga Forward	TGCAGTGGTTAGGGTGTCG
Oga Reverse	AGCAAACGCTGGAACTCTCC

Supplementary Table S3. Primers used for RT-qPCR analysis.
